# Glucagon-like Peptide-1 Receptor Agonists in the Management of Type 2 Diabetes Mellitus and Obesity: The Impact of Pharmacological Properties and Genetic Factors

**DOI:** 10.3390/ijms23073451

**Published:** 2022-03-22

**Authors:** Jasna Klen, Vita Dolžan

**Affiliations:** 1 Department of Abdominal Surgery, Division of Surgery, University Medical Centre Ljubljana, 1000 Ljubljana, Slovenia; jasna.klen@kclj.si; 2 Pharmacogenetics Laboratory, Institute of Biochemistry and Molecular Genetics, Faculty of Medicine, University of Ljubljana, 1000 Ljubljana, Slovenia

**Keywords:** type 2 diabetes mellitus, obesity, incretins, GLP-1 receptor agonists, cardiovascular safety, genetic polymorphisms

## Abstract

Glucagon-like peptide-1 (GLP-1) receptor agonists are a new class of antihyperglycemic drugs that enhance appropriate pancreatic β-cell secretion, pancreatic α-cell (glucagon) suppression, decrease liver glucose production, increase satiety through their action on the central nervous system, slow gastric emptying time, and increase insulin action on peripheral tissue. They are effective in the management of type 2 diabetes mellitus and have a favorable effect on weight loss. Their cardiovascular and renal safety has been extensively investigated and confirmed in many clinical trials. Recently, evidence has shown that in addition to the existing approaches for the treatment of obesity, semaglutide in higher doses promotes weight loss and can be used as a drug to treat obesity. However, some T2DM and obese patients do not achieve a desired therapeutic effect of GLP-1 receptor agonists. This could be due to the multifactorial etiologies of T2DM and obesity, but genetic variability in the GLP-1 receptor or signaling pathways also needs to be considered in non-responders to GLP-1 receptor agonists. This review focuses on the pharmacological, clinical, and genetic factors that may influence the response to GLP-1 receptor agonists in the treatment of type 2 diabetes mellitus and obesity.

## 1. Introduction

Type 2 diabetes mellitus (T2DM) is a heterogeneous, chronic, progressive metabolic disease accounting for 90–95% of all diabetes. T2DM presents a severe public health problem with a global prevalence of 6059 cases per 100,000 T2DM. Male patients show a slightly higher prevalence than females. Patients with T2DM have insulin resistance, and genetically predisposed insulin deficiency clinically manifests as hyperglycemia. Patients with T2DM are usually overweight and obese [1,2]. Obesity is defined as a body mass index (BMI) of 30 kg/m^2^ or higher. It is associated with insulin resistance, hyperinsulinemia, endothelial dysfunction, inflammation, and prothrombotic state. Obesity is a significant risk factor for the occurrence and mortality of T2DM, hypertension, cardiovascular diseases, non-alcoholic steatohepatitis, obstructive sleep apnea, orthopedic problems, mental health complications such as anxiety and depression, and cancer. The word “diabesity” is being used to characterize the combined negative health impacts of obesity and diabetes [3].

T2DM and obesity have multifactorial etiologies, and both genetic and environmental factors play a role in their pathogenesis. Genetic factors account for 30–40% of body mass index (BMI) variation, whereas the environment accounts for 60–70%. Gene–environment interactions thus play an essential role that needs to be better understood. Some people are genetically predisposed to obesity, but their genotype may only manifest in combination with exposure to certain environmental factors, such as high-fat diets and sedentary lifestyles. Low adjusted sedentary energy expenditure, high respiratory quotient (RQ; carbohydrate-to-fat oxidation ratio), and low degree of spontaneous physical activity have all been shown to predict weight gain. Insulin sensitivity was inversely associated with BMI in a group of non-diabetic people with a wide range of BMI. Obese persons require more insulin than non-obese ones to maintain normal glucose tolerance in fasting and postprandial states. The main goals of the treatment of obesity are the prevention or treatment of related complications and concomitant diseases, the achievement of well-being, the fight against stigma, and a positive body image and self-image. It was proposed that favorable results can be achieved by reducing body weight by 5–15% [4].

## 2. Incretin System

It was shown that oral glucose stimulates insulin secretion more than intravenous glucose infusion, even when the same plasma glucose concentration profiles are achieved. This is the so-called incretin effect. Incretins, such as glucagon-like peptide-1 (GLP-1) and glucose-dependent insulinotropic polypeptide (previously called gastric inhibitory polypeptide) (GIP), are part of the endocrine system and are involved in postprandial stimulation of insulin secretion and the physiological regulation of glucose homeostasis [5].

GLP-1 is a 37 amino acid long polypeptide secreted from specialized L cells primarily located in the brush border of the small and the large intestine. Small amounts of GLP-1 are continuously cleaved from proglucagon and excreted, but excretion rises rapidly after mixed meal ingestion. Degradation of GLP-1 is mediated by the enzyme dipeptidyl-peptidase 4 (DPP-4) in the portal vein and liver. Only 1015% of GLP-1 enters the systemic circulation. GLP-1 is also secreted as a neurotransmitter from neurons in the brainstem that project in areas related to the regulation of energy homeostasis [6].

GLP-1 acts through a receptor (GLP-1R) that belongs to the B subfamily of the G protein-coupled receptor family. It consists of seven transmembrane domains in the form of alpha-helices, which are interconnected by six loops, of which three are intra- and three extracellular [7]. GLP-1 first contacts the N-terminal domain and then all three extracellular loops of GLP-1R [8]. Signal transmission operates via a signaling cascade that ultimately increases the cell’s cyclic adenosine monophosphate (cAMP) [7].

GLP-1 receptors (GLP-1R) are expressed not only on pancreatic β-cells but also on a subset of pulmonary epithelial cells, atrial cardiac myocytes, cells lining the gastric pits and small intestinal mucosa, and neurons in several brain regions. They are also located on the nodular ganglion of the vagal nerve, whose central branches terminate in the solitary nucleus in the brainstem. Information from the nucleus is transmitted to the hypothalamus, thalamus, and hemispheres [6,9].

The secretion of incretins is controlled by a series of integrated mechanisms that include direct interaction with distinct chemosensors on the brush border of K and L cells in the gut and multiple indirect neuro–immuno–hormonal loops [10].

GLP-1 increases the synthesis and release of insulin from pancreatic β-cells. GLP-1 regulates the gene expression of pancreatic β-cells, inhibits apoptosis of β-cells, protects them from glucolipotoxicity, and improves their function. In addition, GLP-1 also reduces glucagon secretion by pancreatic α-cells. This reduces glucose production in the liver, reflected in lower blood glucose levels. GLP-1 may additionally act through the activation of visceral afferent neurons. On the other hand, it centrally regulates food intake, energy consumption, and the functioning of the digestive system, thus slowing down gastric emptying and reducing acid secretion, resulting in decreased appetite and body weight. GLP-1 also lowers blood lipoproteins, systolic blood pressure, and inflammation [11,12].

According to meta-analyses, there are no systematic differences in nutrient-induced GLP-1 secretion between healthy and T2DM subjects despite significant inter-individual variance in secretory responses. However, patients with advancing stages of T2DM may have reduced levels of active GLP-1. On the other hand, physiological and pharmaceutical GLP-1 concentrations cause insulinotropic (and glucagonostatic) effects in patients with T2DM [10]. 

Obesity has been shown to reduce the incretin effect even in the absence of impaired glucose tolerance or T2DM [10]. Novel therapeutic approaches for T2DM and obesity treatment rely on the incretin effect of GLP-1R agonists on both pancreatic β-cells and other peripheral and central mechanisms of action.

## 3. Pharmacological Properties of GLP-1 Receptor Agonists

GLP-1 receptor agonists (GLP-1 RAs) are polypeptides injected subcutaneously, similar to insulin. According to their structure, they can be grouped into exenatide and human-like GLP-1. Depending on the time of receptor activation, they are divided into two groups. Short-acting compounds provide short-term activation of the GLP1 receptor, they reach peak concentration in a few hours, and after 6–10 h, their concentrations fall sharply or even to zero. Long-acting compounds activate the receptor continuously, for a long time, and can be administered once a day or once a week [13].

### 3.1. Short-Acting GLP-1 RAs

Exenatide was the first incretin analog approved for clinical use, and it has also been the longest studied. Exenatide shares approximately 50% of the amino acids with human GLP-1 and has a similar affinity for GLP-1 receptors. Due to its half-life (t ½) of 2.4 h, it should be administered twice daily. It is mainly excreted through the kidneys. Early research has shown that after 30 weeks of treatment twice daily, the therapy improves fasting and postprandial glucose levels [13].

The second short-acting GLP-1 RAs was lixisenatide, which can be administered daily. It is designed as a C-terminal modification with six lysine residues and the absence of one proline, allowing physiological degradation by DPP-4. The drug binds strongly to the receptor. Approximately 55% of lixisenatide is bound to proteins. Its half-life (t ½) is 3 h. Lixisenatide is eliminated by glomerular filtration followed by tubular reabsorption [13].

### 3.2. Long-Acting GLP-1 RAs

The long-acting agonist liraglutide is a modified form of GLP-1 obtained by replacing the amino acid serine with arginine and adding the C16 palmitoyl fatty acid side chain on lysine binding to serum albumin and thus increases resistance to degradation by DPP-4. Following subcutaneous injection of liraglutide, plasma concentrations are stable for up to 13 h after administration once a day, and 24-h blood glucose monitoring is achieved. More than 98% of liraglutide is bound to plasma proteins. Liraglutide is metabolized similarly to large proteins, so no organ is considered the primary route of excretion [13].

Exenatide LAR is released biphasically from microparticles. The initial fast peak is followed by a slower release. It can be administered independently of food intake and meals at any time of the day. The full therapeutic effect is achieved in two weeks [13].

Dulaglutide consists of two identical GLP-1 analog peptide chains, approximately 90% homologous to native human GLP-1 and linked to the heavy chain of the immunoglobulin G4 (IgG4). The addition of IgG4 increases the peptide’s size, which helps reduce the rate of renal clearance. At the same time, the Fc fragment (crystallizable region) of IgG4 prevents the formation of antibodies and reduces the potential for immune cytotoxicity. Peak plasma concentrations are reached within 48 h. It is degraded via the general protein breakdown pathways into amino acids [13].

Semaglutide is another long-acting GLP-1 RA. In healthy adults, subcutaneous administration of 0.5 mg semaglutide reached the maximum concentration (Cmax) in 24–56 h [14,15]. Semaglutide has 94% amino acids identical to native GLP-1. Still, it has three-tier modifications that make it less susceptible to degradation. In this way, its half-life is extended by one week to be used once a week. As more than 99% of semaglutide is bound to plasma albumin, systemic removal is slower, and absorption is delayed. Semaglutide had a greater volume of distribution (0.102 L/kg) than liraglutide and a slower body clearance (0.0016 L/h/kg) [15]. Semaglutide is metabolized by proteolytic cleavage of the peptide backbone and b-oxidation of the fatty acid side chain to six different metabolites, primarily excreted in urine and feces. On the other side, the excretion of the intact drug in the urine is minimal, so renal dosing is not necessary [14,15].

Efpeglenatide is an exendin analog containing single amino acid modification linked to a crystallizable (Fc) fragment of human immunoglobulin G for slower clearance and once-weekly dosing. The geometric mean t ½ varies between 135 and 180 h. In the single dosage study, the maximum serum concentration (t_max_) of efpeglenatide ranged from 72 to 144 h. Compared to liraglutide and dulaglutide, efpeglenatide is associated with faster dissociation from the GLP-1R and allows more cell surface receptors to remain available for signaling. Efpeglenatide leads to a more significant accumulation of cyclic adenosine monophosphate and insulinotropic activity after chronic exposure [16].

**Figure 1:**
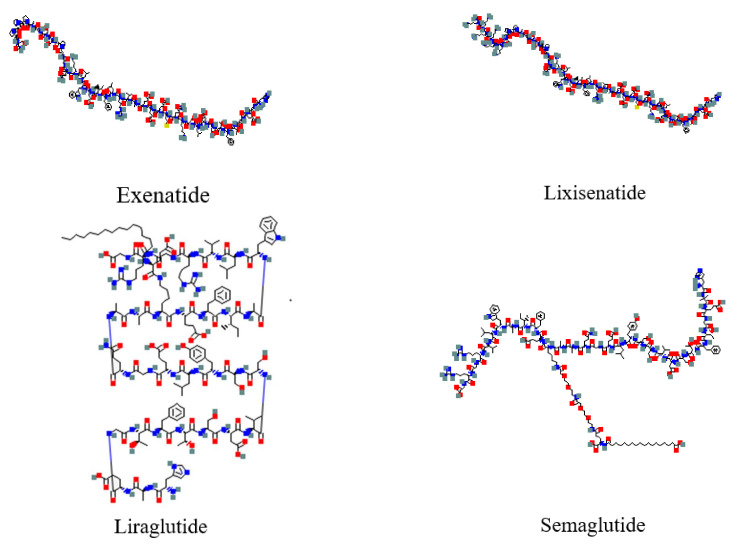

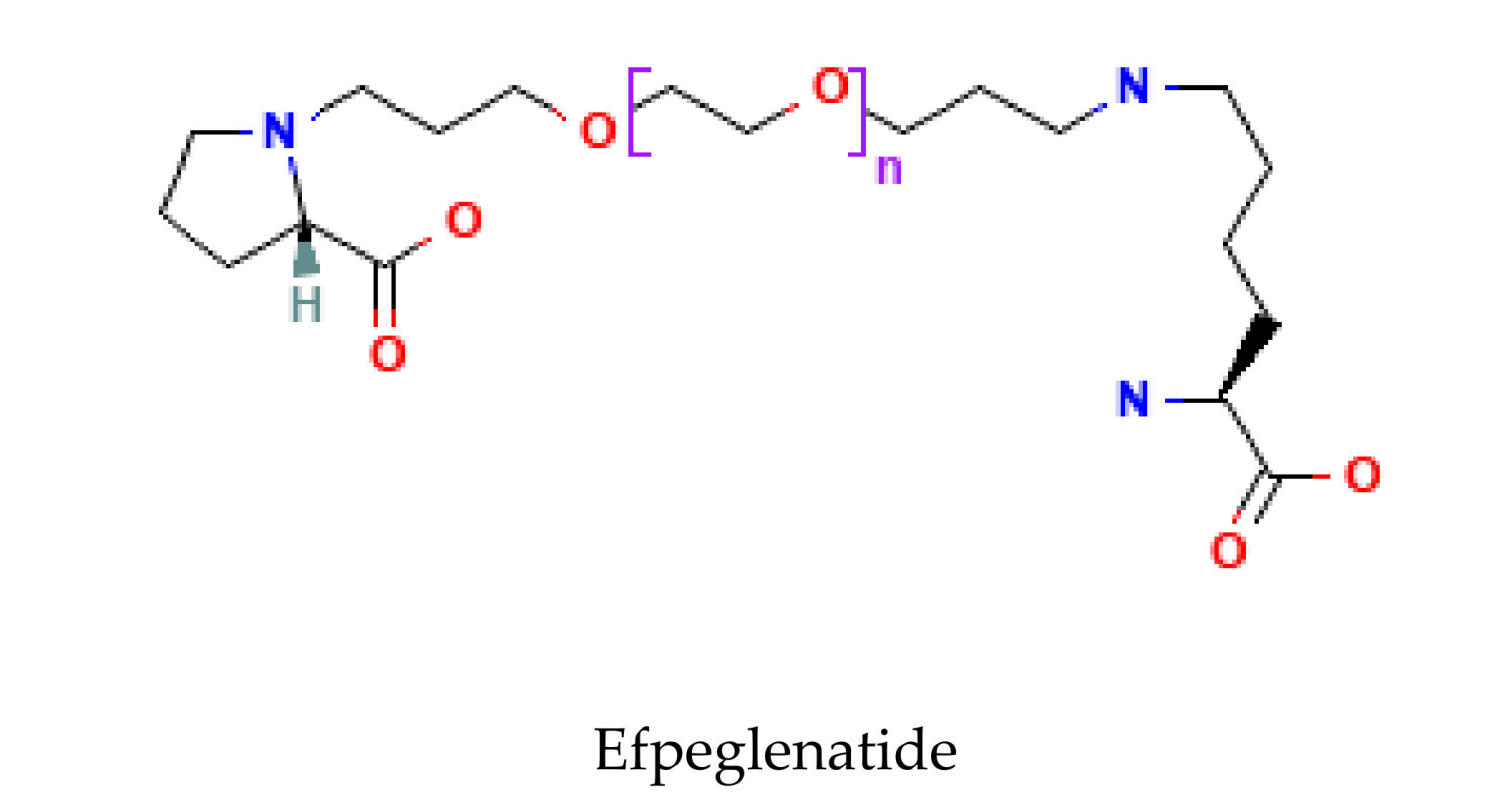
Molecular structure of GLP-1 receptor agonists (https://pubchem.ncbi.nlm.nih.gov/, accessed on 20 February 2022).

The molecular structures of GLP-1 RAs are shown in Figure 1.

## 4. Clinical Efficacy of GLP-1 RAs

Differences in the duration of action of GLP-1 RAs lead to differences in their effect on blood glucose regulation. Short-acting GLP-1 RAs more strongly delay gastric emptying, resulting in a more significant impact on postprandial blood glucose. On the other hand, the longer half-life of long-term GLP-1 RAs allows better regulation of glucose concentrations, including fasting plasma glucose. If GLP-1 RAs are administered as monotherapy, they can reduce the HbA1c levels by 0.7–1.51. In combination with other oral antihyperglycemics or as part of triple therapy, further reduction in HbA1c levels by 0.4–1.9 may be reached. In addition to the beneficial effect on glucose levels, body mass may also decrease by an average of 0.2–4 kg. The highest levels of body mass reduction are achieved in patients with a body mass index (BMI) above 30 kg/m^2^ [13].

## 5. Side Effects of GLP-1 RAs

The most common side effects, which usually occur immediately upon initiation of therapy, are nausea (8–44%), vomiting (4–18%), and diarrhea (6–20%). It is not yet clear whether these side effects impact digestive function or have indirect effects on the central nervous system, either through access to the brain through areas without a blood–brain barrier or indirectly through receptors on branches of afferent parasympathetic nerves. Nausea and vomiting are less common when using prolonged-release forms. Although they belong to the same group, side effects also depend on the composition, dose, and basic antihyperglycemic therapy (metformin) [17]. GLP-1 RAs are associated with a very low risk of hypoglycemia, but caution should be exercised when used in combination with other antihyperglycemic agents, such as sulfonylurea [18].

T2DM patients are three times more likely to develop acute pancreatitis than healthy individuals, but the mechanisms have not been elucidated. In mouse models, they found that GLP-1 RAs increase acinar cell mass in the pancreas, enhancing amylase and lipase synthesis [19]. The ELIXA (Evaluation of LIXisenatide in acute Coronary Syndrome) study showed that the number of acute pancreatitis in the lixisenatide group was lower than in the placebo group [20]. However, acute pancreatitis has been reported in post-marketing studies in individual exenatide users. T2DM patients with semaglutide and dulaglutide have elevated pancreatic enzymes [19]. Pancreatic malignancy occurred in studies with exenatide and placebo groups in equal numbers. GLP-1 RAs were associated with a lower risk of prostate, lung, and colon cancer, but a higher risk of thyroid cancer [21]. FDA has recommended that patients at increased risk for medullary adenoma or thyroid adenocarcinoma and with multiple endocrine neoplasia syndrome 2 should not be treated with GLP-1 RAs [22]. GLP-1 receptors were found to be expressed in neoplastic and hyperplastic lesions of thyroid C cells and on follicular derived papillary thyroid cancer cells [23]. However, dulaglutide treatment was not found to increase calcitonin levels in the reported case of a patient with pre-existing medullary thyroid cancer [24]. Although the cardiovascular outcomes trial did not find an association between GLP-1 RAs and thyroid cancers [25], the recent FDA Adverse Event Reporting System (FAERS) database analysis confirmed a higher risk of thyroid cancer [21]. Among other side effects, semaglutide treatment was associated with more vitreous hemorrhages, blindness, or conditions that required photocoagulation therapy (*p* = 0.2) [14].

## 6. Cardiovascular Benefits and Outcomes

Patients with T2DM are two to four times more likely to develop cardiovascular diseases and these events usually occur at a younger age. According to the latest data, we can conclude that the prevalence of cardiovascular and atherosclerotic cardiovascular diseases is 34.8% (95% CI = 32.7–36.8) and 31.8% (95% CI = 29.7–33), respectively [26].

Native GLP-1 was reported to have cardioprotective effects because of GLP-1 expression in the vascular endothelium and the myocardium. In animal studies, deficiency of GLP receptors resulted in decreased resting heart rate (HR) and impaired left ventricular (LV) contractility. Infusion of GLP-1 improved LV contractility in dilatative cardiomyopathy and hypertension and endothelial function because of its diuretic and natriuretic effects. GLP-1 also has a beneficial impact on myocardial viability and vasodilative outcomes independent from or dependent on nitric oxide [27].

In patients with T2DM, GLP-1 RAs liraglutide and exenatide reduced blood pressure (BP) in the range of 1–5 mmHg, but mechanisms are unknown [27]. In a study with overweight males without chronic diseases, Smits et al. found increased HR and systolic BP after intravenous administration of exenatide [28]. Another study found a significant increase in the mean cardiac output, a reduced total peripheral resistance, and a significantly reduced urinary sodium. This data suggested that reflex tachycardia may be a consequence of reduced total peripheral resistance [29].

GLP-1 RAs also have anti-inflammatory and potentially anti-atherosclerotic effects. In T2DM patients with cardiovascular diseases (CVD), plasminogen activator inhibitor 1 (PAI-1) is elevated and correlates with atherosclerosis. Compared to placebo, liraglutide administration decreased PAI-1 and B-type natriuretic peptide (BNP) levels [30]. Decreased pro-inflammatory markers may impact the inflammatory cascade, which leads to atherosclerosis [31]. Exenatide reduced hs-CRP, endothelin-1 levels, and triglyceride concentrations (−0.43 mmol/L) [32,33] and elevated high-density lipoprotein cholesterol concentrations (0.12 mmol/L) in patients with T2DM. However, favorable changes in lipid profile were not attributed to improved adiposity, which is often associated with a low-grade inflammation [34].

Intima-media thickness (ITM) is associated with atherosclerosis [35]. In patients with T2DM without CVD treated with liraglutide, carotid IMT decreased from 1.19 ± 0.47 to 0.94 ± 0.21 mm (*p* < 0.01) [36]. On the other hand, in severely obese patients with longstanding T2DM and comorbidities, there is no change in IMT despite good glycemic control and reduction in adipose tissue [37]. Intravenous GLP-1 infusion also improves global and regional wall LV function in patients with good LV function and coronary artery disease [38]. Patients with T2DM and acute myocardial infarction (MI) liraglutide could prevent the progression of post-MI LV remodeling [39].

The ELIXA study included patients with T2DM who had previously suffered an acute myocardial infarction or had been hospitalized for unstable angina pectoris 180 days before inclusion. In addition to standard therapy, they received lixisenatide or a placebo. The primary composite endpoints are cardiovascular death, myocardial infarction, stroke, or hospitalization for unstable angina. Major cardiovascular adverse events (MACE) occurred in 13.4% of the lixisenatide group. In any case, they did not demonstrate the superiority of lixisenatide compared to placebo (*p* = 0.81). There were no differences between the groups in the number of patients treated for heart failure or in the number of deaths (HR = 0.94; 95% CI = 0.78–1.13). It also had a beneficial effect on reducing proteinuria, independent of the HbA1c baseline [20,40].

In the EXSCEL (Exenatide Study of Cardiovascular Event Lowering) study, MACE was slightly more frequent in the placebo group. In addition, long acting exenatide was non-inferior to placebo (*p* < 0.001) and also not superior to efficacy (*p* = 0.06). There were no statistically significant differences between the groups regarding cardiovascular mortality, fatal and non-fatal myocardial infarction, non-fatal or fatal stroke, inpatient treatment for heart failure, and acute coronary events [41].

In the LEADER (Liraglutide Effect and Action in Diabetes: Evaluation of Cardiovascular Outcome Results) study, significantly fewer cardiovascular events occurred in the liraglutide group compared to placebo, namely 13.0% (*p* < 0.001 for non-inferiority, *p* = 0.01 for superiority). Fewer also died due to a cardiovascular event (*p* = 0.007) or any other cause (*p* = 0.02). Non-fatal myocardial infarction, stroke, and hospital treatment for heart failure were also fewer, but not significantly. The number of microvascular complications (nephropathy, retinopathy) was lower in the liraglutide group (HR = 0.85; 95% CI = 73–0.97, *p* = 0.02) [18].

The longest REWIND (Researching Cardiovascular Events with a Weekly Incretin in Diabetes) study to date included only 31% of patients with known cardiovascular disease. Dulaglutide or placebo was added to the existing standard therapy. The primary endpoint was again the first occurrence of MACE, while the secondary endpoint was in addition to all components of the primary endpoint and included the occurrence of nephropathy or retinopathy, hospital treatment for unstable angina pectoris, heart failure, and death from all causes. In patients with or without pre-existing cardiovascular disease, a reduced number of cardiovascular events was demonstrated in the dulaglutide group [42].

The SUSTAIN-6 (Semaglutide Unabated Sustainability in Treatment of Type 2 Diabetes) study included 3.297 patients with T2DM, of whom 83% had pre-existing cardiovascular disease, chronic renal failure, or both. For 104 weeks, they received semaglutide or placebo once weekly in addition to their regular therapy. Three-point MACE occurred in 6.6% of patients receiving semaglutide (*p* < 0.001 for non-inferiority). Approximately the same percentage of patients suffered a myocardial infarction and cardiovascular death (*p* = 0.12). In the placebo group, more patients (2.7%) suffered from stroke (*p* = 0.04), and there were fewer acute renal failure or acute exacerbations of chronic renal failure [14].

AMPLITUDE-O was a randomized, placebo-controlled study that evaluated efpeglenatide versus placebo in patients with T2DM with cardiovascular or kidney disease and one or more other cardiovascular risk factors. They found a particular reduction in hospital admissions for heart failure [43].

An overview of studies assessing cardiovascular and renal safety of GLP-1 RAs is presented in Table 1.

## 7. Obesity and GLP-1 Receptor Agonists

The incretin effect is decreased in obesity regardless of impaired glucose tolerance or T2DM. This is probably a consequence of the reduced contribution of GLP-1 to insulin secretory responses [10]. Leptin is a hormone released from white adipose tissue. In obese patients, leptin resistance is key to reduced GLP-1 levels [44]. In the mouse model, the arcuate nucleus inside the hypothalamus, the area postrema, and the nucleus tractus solitarii all have a role in the influence of systemically administered GLP-1 RAs on appetite, satiety, calorie intake, and body weight. In this model, GLP-1 RAs reduced the activity of neuropeptide Y/agouti-related peptide (NPY/AgRP) neurons and prevented meal start. The arcuate nucleus of the hypothalamus and the brain stem sends inhibitory signals to the lateral parabrachial nucleus. These signals are disinhibited by POMC/CART neurons that express GLP-1 receptors and directly or indirectly activate parabrachial neurons and repress NPY/AgRP neurons [45].

The capacity of GLP-1 RAs to impact food choices has also been proven, and there is no evidence that semaglutide treatment interfered with the reduction in energy expenditure that typically occurs with weight loss in human trials. Patients who are treated with semaglutide derive less pleasure from eating. This observation aligns with magnetic resonance imaging where GLP-1 receptor activation decreased anticipatorily and increased consummatory meal reward [45].

After longer-term administration, all GLP-1 RAs were associated with weight reduction, but to varying degrees. The highest weight reduction was achieved in clinical trials with semaglutide 2.4 mg once weekly, administered subcutaneously or taken orally. The difference in body weight between baseline and week 68 in the semaglutide group (2.4 mg) was −15.3 kg, compared to −2.6 kg in the placebo group in the STEP 1 clinical trial [46]. Liraglutide 3 mg daily was also licensed for pharmacological therapy of obesity at doses slightly higher than those used for T2DM treatment. After 56 weeks of treatment with liraglutide, 33 % of obese patients lost 10 % of weight, while 14 % of obese patients achieved 15 % weight loss [47]. A recent meta-analysis found that liraglutide (3.0 mg) treatment resulted in an average weight loss of 5.25 (6.17 to 4.32) kg when compared to placebo in patients without T2DM [48].

Clinical trials with liraglutide showed that patients lost less weight and reached a plateau quickly [47,48]. Following weight loss induction, GLP-1 RAs should be administered continuously as body weight may return to or near to baseline levels within a few months after discontinuation. However, patients on GLP-1 RAs may achieve long-term weight loss if they are very motivated, physically active, and have optimized nutrition [49].

In everyday clinical practice, we, however, observe that some T2DM patients do not achieve a reduction in body weight with GLP-1 RAs [45]. This could be due to the multifactorial etiologies of T2DM and obesity, including environmental and genetic factors. Therefore, a multidisciplinary approach is needed to address both lifestyle modifications and pharmacological treatment. However, genetic factors need to be considered as well as some patients may be non-responders to GLP-1 RAs due to genetic variability in the GLP-1 receptor or signaling pathways.

## 8. Genetic Variability of the Incretin System

GLP-1R is encoded by the *GLP1R* gene, located on chromosome 6p21.2 [50]. Various changes may contribute to genetic variability in the *GLP1R* region, particularly in the coding regions such as exons and exon–intron boundaries, and changes in the regulatory regions such as 5′-upstream regulatory regions and promoter and 3’-untranslated regions [51]. 

Mutations in the *GLP1R* gene have been associated with altered GLP-1R function; thus, affecting both postprandial pancreatic β cell insulin secretion and response to receptor agonists. Several *GLP1R* missense variants were found in T2DM patients. Direct sequencing of the entire *GLP1R* coding region in 36 unrelated Japanese patients with T2DM identified five missense variants in exons: Pro7Leu (CCG → CTG, rs10305420, exon 1), Arg44His (CGC → CAC, rs2295006, exon 2), Arg131Gln (CGA → CAA, rs3765467, exon 4), Thr149Met (ACG → ATG, rs112198, exon 5), and Leu260Phe (TTA → TTC, rs1042044, exon 7). They have also identified four silent variants in exons: Arg48Arg (CGC → CGG, exon 2), Lys130Lys (AAG → AAA, exon 4), Arg176Arg (AGG → CGG, exon 6), and Ile400Ile (ATA → ATC, exon 12). When a larger cohort of 791 Japanese patients with T2DM and 318 control subjects was screened for the missense variants, the frequencies of Pro7Leu, Arg44His, and Leu260Phe did not differ between T2DM patients and healthy controls, nor did they differ between T2DM patients that were insulin users or non-users. Furthermore, no significant differences in body mass index (BMI), age of T2DM onset, fasting insulin levels, or insulin usage were observed among different genotype groups. On the other hand, the Thr149Met variant, although rare and detected in only one T2DM patient but not in controls, impaired glucose effectiveness and decreased insulin and C-peptide secretion [51].

The functional impact of Thr149Met was assessed by transient overexpression in COS-7 and HEK 293 cells, and the variant GLP-1R showed similar expression levels as the wild-type receptor. However, it significantly reduced binding affinities for GLP-1 and exendin-4 [52]. Although mutations in residue 149 significantly decreased receptor functions, the functionality of the receptor could be restored with the small-molecular allosteric agonists. On the other hand, mutations of residue 333 had minimal impact on receptor functions. However, the mutated receptors were less sensitive to allosteric modulation than the wild-type receptor [53].

Besides mutations that significantly affect GLP-1R receptor function, common single-nucleotide polymorphisms (SNPs) occurring in more than 1% of the population were found in the *GLP1R* gene. The four most common *GLP1R* SNPs that lead to amino acid substitution are rs1042044 (Leu260Phe, TTA → TTC), rs10305420 (Pro7Leu, CCG → CTG), rs6923761 (Gly168Ser, GGC → AGC), and rs3765467 (Arg131Gln, CGA → CAA) [54].

### 8.1. Associations of GLP1R Variants with Glycemic Traits and Incretin Effect

Several studies investigated *GLP1R* polymorphisms related to glucose levels and insulin secretion and the pathogenesis of obesity and T2DM diabetes. A pilot study in 88 non-diabetic subjects investigated 21 *GLP1R* tag SNPs. Among them, rs6923761 (Gly168Ser) and rs3765467 (Arg131Gln) were nominally associated with decreased insulin secretion stimulated by hyperglycemia and GLP-1 infusion [54]. A mouse ß-cell model system study showed that besides rs3765467 (Arg131Gln), rs10305492 (Ala316Thr) also significantly reduced glucose-stimulated insulin secretion. Furthermore, these SNPs also decreased β cells viability and mass by promoting β cell apoptosis [55].

The most extensive analysis of associations of exonic SNPs with glycemic traits in non-diabetic individuals of European and African ancestries was performed on combined data from 23 large genome-wide association studies with available information on fasting glucose (n = 60,564) and insulin levels (n = 48,118). The exome data were obtained from studies using the HumanExome BeadChip that allowed the capture of common SNPs with minor allele frequencies (MAF) above 5 % and rare single nucleotide variants (SNVs) with MAF below 1%. The data analysis revealed the association of a rare *GLP1R* variant rs10305492 (Ala316Thr) with lower fasting glucose levels, but not with fasting insulin levels or incretin response in a combined sample of subjects of European and African ancestries. Additionally, the associations with T2DM risk were investigated on data from 16,491 T2DM and 81,877 control subjects; however, no associations were found for *GLP1R* variants [56].

Incretins were also shown to play a role in the regulation of gastric emptying [57]; however, the studies investigating the associations of *GLP1R* SNPs on the rate of gastric emptying are scarce, and their results were not consistent. A study that included 48 healthy Caucasian males genotyped for 27 Tag SNPs in the *GLP1R* region reported significant associations of several tag SNPs with the gastric emptying rate after oral ingestion of a glucose solution. The maximal gastric emptying rate was significantly higher in subjects with rs742764 CC genotype when compared to TT and TC genotypes. On the other hand, the maximal gastric emptying rate was considerably slower in subjects with rs2254336 AA genotype than the TT and TA genotypes. *GLP1R* rs9283907, rs2268657, and rs2254336 were also associated with differences in half-emptying time as well as the maximal emptying rate [58].

A more recent study that investigated two functional *GLP1R* SNPs, namely rs6923761 and rs1042044 in 51 participants from a more extensive study that consented to genotyping, observed no significant effect of these SNPs on gastric emptying, plasma glucose, and GLP-1 concentrations after enteral infusion of dextrose [59].

### 8.2. Associations of GLP1R Variants with Obesity and T2DM

Several studies investigated *GLP1R* polymorphisms in relation to the pathogenesis of obesity and T2DM; however, the results were not consistent among studies.

Tokuyama et al. observed no associations between five missense and four silent *GLP1R* variants with either BMI in Japanese T2DM patients and healthy controls or with T2DM risk. Furthermore, *GLP1R* variants had no impact on the requirement for insulin usage among T2DM patients [51].

An extensive cross-sectional survey that investigated associations of *GLP1R* rs6923761 with anthropometric parameters and components of metabolic syndrome included a sample of 1122 Spanish obese subjects. Body mass index, weight, fat mass, waist circumference, and waist to hip ratio were higher in the rs6923761 GG genotype when compared to GA or AA genotype carriers with and without metabolic syndrome. However, no associations were observed with the risk for metabolic syndrome [60]. Associations of *GLP1R* rs6923761 polymorphism with the anthropometric parameters and metabolic traits were investigated in more detail in 341 obese subjects from the Spanish region of Castilla y Leon. Significantly higher BMI, weight, fat mass, waist to hip ratio, and waist circumference, as well as higher insulin levels and higher HOMA-IR index values, were observed in the group of subjects with the wild-type rs6923761 GG genotype as compared to carriers of one or two polymorphic A alleles [61].

Associations with T2DM risk were investigated in a study that included 150 T2DM patients and 150 healthy controls from Egypt. This study found no association of *GLP1R* rs367543060 polymorphism with T2DM, although they have observed significantly lower GLP-1 levels in diabetic patients than controls [62].

Targeted exome sequencing of six genes encoding drug targets for obesity or T2DM in 11,806 individuals with well-defined metabolic traits, followed by targeted genotyping of 39,979 individuals, reported an association of *GLP1R* rs10305492 (Ala316Thr) with lower fasting glucose and T2D risk. In the next stage, they included 61,846 patients with coronary heart disease and 163,728 controls, and they have observed the protective effect of the rs10305492 polymorphism against heart disease. This observation suggested that it is not likely that treatment with GLP-1 RAs would be associated with increased cardiovascular risk [63].

### 8.3. Associations of GLP1R Variants with Cardiovascular Risk Factors

As clinical trials demonstrated a protective effect of GLP-1 RAs on the cardiovascular safety in patients with T2DM [14,64] and GLP-1R was reported to be widely expressed in the cardiovascular system [65], several studies set out to investigate if genetic variability of GLP1R is associated with cardiovascular complications among patients with T2DM.

A prospective study that included 104 Spanish treatment-naïve T2DM patients with diabetes mellitus type 2 observed no effect of *GLP1R* rs6923761 on anthropometric parameters, metabolic traits, or cardiovascular risk factors. However, the basal GLP-1 levels were higher in A allele carriers when compared to non-carriers [66]. The previously mentioned study investigating the associations between *GLP1R* rs6923761 and anthropometric parameters in Spanish obese patients also investigated metabolic factors associated with higher cardiovascular risk. Besides *GLP1R* rs6923761 associations with worse anthropometric factors that may, per se, lead to increased risk of cardiovascular disease, they have also observed significantly lower HDL cholesterol levels and higher triglyceride levels among subjects with the wild-type rs6923761 GG genotype when compared to carriers of one or two polymorphic A alleles [61].

The impact of genetic variability on cardiovascular disease was also studied in T2DM patients from the Chinese Han population. When the allele and genotype frequencies of 11 tag SNPs across the *GLP1R* region were assessed in 394 T2DM patients with and 217 patients without coronary artery disease, the rs4714210 GG genotype was associated with lower cardiovascular disease risk and this association remained significant also after adjustment for other known risk factors for cardiovascular disease [67].

### 8.4. Associations of GLP1R Variants with Body Weight Lowering Potential of Dietary Interventions in Obese Subjects

The effects of *GLP1R* polymorphisms on body weight lowering potential were also evaluated in dietary interventions.

Four consecutive studies in Spanish obese patients have repeatedly shown an association of the polymorphic rs6923761 A allele with anthropometric parameters. However, they failed to demonstrate associations with weight loss in different dietary interventions [68,69,70,71].

First, 280 obese subjects were randomized to a diet low in carbohydrates or a diet low in fats; no association was observed between rs6923761 polymorphism and weight loss after 5 months of either hypocaloric diet. However, anthropometric parameters such as fat mass and waist circumference were lower in polymorphic rs6923761 A-allele carriers than non-carriers [68].

Similar observations were reported in a study that evaluated the combined effects of *GLP1R* rs6923761 polymorphism on weight loss and anthropometric and metabolic parameters associated with cardiovascular risk factors in 391 obese patients randomized to 3 months intervention with hypocaloric diets with either high monounsaturated fat or high polyunsaturated fat content. Despite better anthropometric parameters in carriers of polymorphic rs6923761 A allele than non-carriers, no weight loss associations were observed in any dietary interventions [69].

Another prospective study in Spanish obese patients investigated the effect of *GLP1R* rs6923761 polymorphism on weight loss and metabolic parameters in 91 obese subjects after 3 months of a standard hypocaloric diet. Although pretreatment and posttreatment BMI, weight, fat mass, waist circumference, and triglyceride levels were higher in *GLP1R* rs6923761 wild-type homozygotes than carriers of at least one polymorphic allele, *GLP1R* rs6923761 genotype was not associated with weight loss [70].

*GLP1R* rs6923761 polymorphism was also investigated for associations with cardiovascular risk factors, adipokine levels, and weight loss in 211 obese Spanish subjects randomized to 9 months of intervention with a high-protein/low-carbohydrate or standard hypocaloric diets. The dietary intervention improved anthropometric parameters and decreased systolic blood pressure in both treatment groups. On a high-protein/low-carbohydrate diet, LDL cholesterol, total cholesterol, leptin, insulin levels, and HOMA-R decreased irrespective of the rs6923761 genotype. However, these parameters were only reduced among subjects on a standard hypocaloric diet in patients with wild-type genotype. Again, no associations were observed between rs6923761 genotype and weight loss, irrespective of the nature of dietary intervention [71].

An overview of studies investigating the associations of *GLP1R* polymorphisms with glycemic traits, incretin effect, obesity, and T2DM are presented in Table 2.

### 8.5. Associations of GLP1R Variants with Response to GLP-1 Receptor Agonists

Since GLP-1 RAs exert their biological effects by interacting with the GLP-1 receptor, interindividual differences in the expression of these receptors or *GLP1R* polymorphisms may modify biological responses to treatment with GLP-1RAs. As it has been reported that differences in the insulinotropic response to exogenous GLP-1 in healthy volunteers were associated with *GLP1R* rs6923761 (Gly168Ser) and rs3765467 (Arg131Gln) polymorphisms [54], several studies were set up to establish the relationship between these SNPs and the response to GLP-1 RAs in patients with T2DM.

The impact of *GLP1R* rs6923761 polymorphism on metabolic changes and weight loss secondary to treatment with liraglutide was investigated in 90 overweight Spanish T2DM patients that required initiation of liraglutide treatment because they were not able to achieve good glycemic control with metformin alone. Upon liraglutide treatment, carriers of the polymorphic rs6923761 A allele achieved higher BMI, weight, and fat mass reductions. However, the decrease in basal glucose levels, HOMA-R index, and HbA1c did not differ between both genotypes [72]. Another study investigated the associations of *GLP1R* rs6923761 (Gly168Ser) with the glycemic response to add-on treatment with sitagliptin or vildagliptin in 92 and 48 T2DM patients. Among them, carriers of the rs6923761 polymorphism showed a reduced response to GLP-1 RAs as they achieved significantly lower HbA1c levels after six months of treatment [73].

Regarding the short-acting GLP-1 RA exenatide, a pilot clinical study did not observe any differences in pharmacological effects in response to short-term treatment. The analysis of all 13 *GLP1R* exons and intron–exon junctions in 36 patients with poorly controlled T2DM on combination treatment with insulin and exenatide revealed an association of rs3765467 and rs761386 polymorphisms with clinical response defined as changes in the plasma glucose levels after 3 days of exenatide treatment. However, these differences did not remain significant after adjustments for multiple testing [74].

As the weight loss observed in response to treatment with GLP-1 RAs may be, at least in part, due to their effect on gastric emptying, the impact of *GLP1R* rs6923761 polymorphism on both parameters was investigated in obese T2DM patients from two trials treated with liraglutide for 16 weeks or with exenatide for 30 days, respectively. After treatment with any of the two GLP-1 RAs, carriers of polymorphic *GLP1R* rs6923761 A allele showed a significantly greater delay in gastric emptying than non-carriers. The difference in weight loss did not achieve statistical significance [75].

However, the effect of *GLP1R* rs6923761 on the weight lowering potential of liraglutide was demonstrated in obese women with polycystic ovary syndrome. The study included 57 patients treated with liraglutide genotyped for rs6923761 and rs10305420. Patients who have lost 5% or more of their initial body weight after 12 weeks of treatment were classified as strong responders. *GLP1R* rs10305420 polymorphism was associated with poor treatment response, while carriers of at least one polymorphic rs6923761 allele tended to lose more weight. The relative decreases in fasting and oral glucose tolerance test-stimulated glucose levels were comparable between both groups [76].

An overview of studies investigating the associations of *GLP1R* polymorphisms with responses to treatment with GLP-1 RAs are presented in Table 3.

## 9. Future Perspectives

Treatment with GLP-1 RAs reduced the risk of MACE and its components and composite kidney outcomes, but only 8 % of all antihyperglycemic agents used are GLP-1 RAs. Considering the proven clinical efficacy in glycemic control and weight lowering as well as the cardiovascular benefits and outcomes, many vulnerable patients with T2DM could be switched from other antihyperglycemic agents to GLP-1 RAs [77,78]. However, in clinical practice, some patients may not respond to GLP-1 RAs as expected, and systematic studies that would address these interindividual differences in treatment response are still lacking [45]. Besides lifestyle, also genetic variability may contribute to the observed differences in treatment responses. Several studies have already identified *GLP1R* SNPs, especially rs6923761, as potential genetic biomarkers of treatment response to GLP-1 RAs. However, it must be noted that most studies conducted so far have failed to observe an association between *GLP1R* SNPs and weight loss. More studies are also needed to investigate the potential impact of genetic variability on the occurrence of side effects of treatment with GLP-1 RAs. If validated in future clinical studies, genotyping for *GLP1R* variants could support the early prediction of treatment response and selection of patients in which treatment with GLP-1RAs would have the most significant clinical effectiveness and safety [79].

## 10. Conclusions

GLP-1 RAs are a new class of antihyperglycemic drugs that are effective in the management of T2DM and also have a favorable effect on weight loss. Their cardiovascular and renal safety in T2DM patients has been extensively investigated and confirmed in many clinical trials. Besides the existing non-pharmacological and pharmacological approaches for the treatment of obesity, it was recently shown that GLP-1 RAs promote weight loss and can be used in the pharmacological treatment of obesity. As genetic variability of the *GLP1R* gene was shown to influence the response to treatment with GLP-1 RAs, pharmacogenetic testing may contribute to personalized and, thus, more effective and safer treatment of T2DM and obesity.

## Figures and Tables

**Table 1:** Cardiovascular and renal safety of GLP-1 receptor agonists.

GLP-1 Receptor Agonists				Median Follow Up (Years)		Mortality				
	Comparator	Study	No. of Patients		MACE	Cardiovascular	General	Heart Failure	Renal Failure	Reference
Liraglutide	Placebo	LEADER	9340	3.8	↓	↓	↓	↓	↓	[18]
Lixisenatide	Placebo	ELIXA	6068	2.1	neutral	neutral	neutral	neutral	impact on macroalbumin uria	[20]
Exenatide-LAR	Placebo	EXSCEL	14,752	3.2	↓	↓	↓	neutral	↓	[41]
Dulaglutide	Placebo	REWIND	9901	5.0	↓	↓	neutral	neutral	↓	[42]
Semaglutide	Placebo	SUSTAIN-6	3297	2.1	↓	neutral	neutral	neutral	↓	[14]
Efpeglenatide	Placebo	AMPLITUDE-O	4076	1.8	↓	↓		↓	↓	[43]

MACE—major adverse cardiovascular events.

**Table 2:** An overview of studies investigating the associations of *GLP1R* polymorphisms with glycemic traits and anthropometric and metabolic characteristics of T2DM and obese patients.

*GLP1R* Polymorphisms	Outcomes Studied	Study Population	Main Findings	Reference
*GLP1R* region sequencing; PCR-RFLP validation of Pro7Leu, Arg44His, Arg131Gln, Thr149Met, Leu260Phe, Arg48Arg, Lys130Lys, Arg176Arg, Ile400Ile	T2DM risk, BMI, insulin secretion, insulin usage	36 Japanese T2DM patients, validation: 791 T2DM and 318 control subjects	Thr149Met found in T2DM patients with decreased insulin secretion; no associations with BMI, T2DM risk, or insulin usage	[51]
21 *GLP1R* tag SNPs	insulin secretion stimulated by hyperglycemia and GLP-1 infusion	88 non-diabetic subjects	rs6923761 (Gly168Ser) and rs3765467 (Arg131Gln) nominally associated with decreased insulin secretion stimulated by hyperglycemia and GLP-1 infusion	[54]
HumanExome BeadChip	fasting glucose and insulin levels, incretin effect	60,564 non-diabetic subjects	rs10305492 (Ala316Thr) associated with lower fasting glucose	[56]
HumanExome BeadChip	T2DM risk	16,491 T2DM and 81,877 control subjects	no association with T2DM risk	[56]
27 Tag SNPs	gastric emptying	48 healthy Caucasian males	faster gastric emptying in rs742764 CC vs. TT and TC; slower in rs2254336 AA vs. TT and TA genotypes	[58]
rs6923761 and rs1042044	gastric emptying, plasma glucose, and GLP-1 levels	51 individuals with dyspepsia	no significant associations	[59]
rs6923761	anthropometric parameters, glycemia, and metabolic traits; cardiovascular risk factors	341 Spanish obese subjects	higher BMI, weight, fat mass, waist to hip ratio, waist circumference, triglycerides, insulin levels, HOMA-IR index values, and lower HDL cholesterol in GG vs. GA or AA genotypes	[61]
rs6923761	anthropometric parameters, metabolic syndrome components	1122 Spanish obese subjects	higher BMI, weight, fat mass, waist to hip ratio, waist circumference in GG vs. GA or AA genotypes; no association with the metabolic syndrome	[60]
rs367543060	T2D risk	150 Egyptian T2DM patients and 150 healthy controls	no association with T2DM risk	[62]
Targeted exome sequencing and genotyping	metabolic traits, T2D risk	T2DM risk: 11,806 (sequencing) and 39,979 (genotyping) individuals	rs10305492 (Ala316Thr) associated with lower fasting glucose and T2D risk	[63]
Targeted exome sequencing and genotyping	cardiovascular risk	61,846 patients with coronary heart disease and 163,728 controls	rs10305492 (Ala316Thr) associated with lower CAD risk	[63]
rs6923761	anthropometric parameters, metabolic traits, cardiovascular risk	104 Spanish treatment-naïve T2DM patients	basal GLP-1 levels higher in A allele carriers vs. non-carriers; no associations with other traits or cardiovascular risk factors	[66]
11 tagSNPs	cardiovascular disease risk	Han Chinese T2D patients: 394 with CAD vs. 217 without CAD	rs4714210 GG genotype associated with lower CAD risk	[67]
rs6923761	anthropometric parameters and weight loss after 5 months of a hypocaloric diet	280 obese Spanish patients randomized to a diet low in carbohydrates or low in fats	polymorphic rs6923761 A allele associated with better anthropometric parameters, but not weight loss	[68]
rs6923761	anthropometric and metabolic parameters of cardiovascular risk after 3 months of a hypocaloric diet	391 obese Spanish patients randomized to a diet with high monounsaturated fat or high polyunsaturated fat content	polymorphic rs6923761 A allele associated with better anthropometric parameters, but not weight loss	[69]
rs6923761	metabolic responses and weight loss after 3 months of a hypocaloric diet	91 obese Spanish patients on a hypocaloric diet	polymorphic rs6923761 A allele associated with better anthropometric parameters and triglyceride levels, but not weight loss	[70]
rs6923761	cardiovascular risk factors, adipokine levels, and weight loss after 9 months of a hypocaloric diet	211 obese Spanish patients randomized to a high protein/low carbohydrate versus standard hypocaloric diet	polymorphic rs6923761 A allele associated with better anthropometric parameters, but not weight loss	[71]

**Table 3:** An overview of studies investigating *GLP1R* polymorphisms and responses to treatment with GLP-1 receptor agonists.

*GLP1R* Polymorphisms	Outcomes Studied	Study Population	Main Findings	Reference
rs6923761	metabolic changes and weight loss after liraglutide add-on treatment	90 overweight Spanish T2DM patients on liraglutide add-on treatment	carriers of polymorphic rs6923761 A allele achieved higher reductions in BMI, weight, and fat mass; no association of rs6923761 genotypes with the decrease in basal glucose levels, HOMA-R index, and HbA1c	[72]
rs6923761	glycemic response after 6 months of gliptin treatment	T2DM patients on sitagliptin (n = 92) or vildagliptin (n = 48)	significantly lower reduction in HbA1c levels in carriers of rs6923761 polymorphism	[73]
all exons and intron-exon junctions	plasma glucose levels after 3 days of exenatide co-treatment	36 patients with poorly controlled T2D on combination treatment with insulin and exenatide	no associations with rs3765467 and rs761386 after adjustment for multiple testing	[74]
rs6923761	gastric emptying and weight loss	obese individuals on liraglutide (n = 20) for 16 weeks or exenatide (n = 40) for 30 days	significantly greater delay in gastric emptying in rs6923761 A-allele carriers compared to non-carriers; no association with weight loss	[75]
rs6923761, rs10305420	weight loss, glycemic control, insulin resistance after 12 weeks of liraglutide	57 obese women with polycystic ovary syndrome treated with liraglutide for 12 weeks	rs10305420 polymorphism associated with poor treatment response, while carriers of at least one polymorphic rs6923761 allele tended to lose more weight	[76]

## Data Availability

Not applicable.
